# Compatibility of Stabilized Whole Blood Products with CD4 Technologies and Their Suitability for Quality Assessment Programs

**DOI:** 10.1371/journal.pone.0103391

**Published:** 2014-08-08

**Authors:** Tao Ding, Michèle Bergeron, Peggy Seely, Xuefen Yang, Tamsir O. Diallo, Margot Plews, Paul Sandstrom, T. Blake Ball, Adrienne F. A. Meyers

**Affiliations:** 1 National Laboratory for HIV Immunology, Public Health Agency of Canada, Ottawa, Ontario, Canada; 2 Department of Medical Microbiology, University of Manitoba, Winnipeg, Manitoba, Canada; 3 Department of Immunology, University of Manitoba, Winnipeg, Manitoba, Canada; 4 Department of Medical Microbiology, University of Nairobi, Nairobi, Kenya; 5 National Laboratory for HIV Immunology, Public Health Agency of Canada, Winnipeg, Manitoba, Canada; University of Cape Town, South Africa

## Abstract

**Background:**

CD4 T cell enumeration is the most widely used prognostic marker for management of HIV disease. Internal quality control and external quality assessment (EQA) programs are critical to ensure reliability of clinical measurements. The utility of stabilized whole blood products (SWBP) as a test reagent for EQA programs such as Quality Assessment and Standardization for Immunological measures relevant to HIV/AIDS (QASI) program have been demonstrated previously. Since then, several new commercial SWBPs and alternative CD4 enumeration technologies have become available. Seven SWBPs were evaluated on seven different enumeration platforms to determine which product(s) are most suitable for EQA programs that support multiple analytical technologies.

**Method:**

Assessment of SWBPs was based on two criteria: (1) accuracy of CD4 T cell measurements and; (2) stability under sub optimal storage conditions.

**Results:**

Three SWBPs (*Multi-Check*, *StatusFlow* and *CD4 Count*) showed accurate CD4 T-cell absolute count and percentage values across six of the enumeration platforms. All products retain stability up to 18 days at 21–23°C with the exception of *Multi-Check-hig*h on FacsCount and *Multi-Check-Low* and *StatusFlow-Low* on Pima. One of the products (*CD4 Count*) retained stability for three days on all platforms tested when stored at 37°C.

**Conclusion:**

This study demonstrated that the characteristics of commercially available SWBPs vary across multiple CD4 platforms. The compatibility of testing panels for EQA programs with multiple analytical platforms needs to be carefully considered, especially in large multiplatform CD4 EQA programs. The selection of a suitable cross-platform SWBP is an increasing challenge as more reagents and platforms are introduced for CD4 T-cell enumeration.

## Introduction

By the end of 2012, more than 9.7 million people living with HIV in low- and middle-income countries were receiving antiretroviral therapy (ART) [1]. CD4 T-lymphocytes are the primary targets of HIV and CD4 T-cell counts serve as an indicator to initiate therapy, monitor disease progression, and alter ART drug regimens [2]. Delivery of reliable treatment requires accurate and precise CD4 T-cell counting [3] and the implementation of internal quality control measures and participation in external quality assessment/assurance (EQA) programs are critical to maintain quality testing [4].

In resource-rich countries CD4 T-cell testing is generally performed with multi-laser clinical flow cytometers. These expensive and complex instruments are not suitable for many resource-limited settings. Over the last decade, the increase in magnitude of HIV treatment in resource-poor regions forced a shift from high end flow cytometers toward lower cost technologies including point-of-care (POC) devices designed for use in remote and resource-limited settings [5]. For some existing CD4 enumeration platforms new reagent kits to determine CD4 T-cells as lymphocyte percentages have become available for the assessment of paediatric HIV infected populations.

QASI (Quality Assessment and Standardization for Immunological measures relevant to HIV/AIDS) is an international program of the Public Health Agency of Canada for CD4 T-cell enumeration that was created in 1996 to assist regions with limited resources by providing assessment of laboratory performance and assistance with remedial action [6]. QASI reaches more than 1000 laboratories in 50 countries worldwide, most of which are located on the African continent. Commercial stabilized whole blood products (SWBP) primarily intended for immunophenotyping quality control purposes are used as a quality control testing panels by the QASI program and others. These whole blood-like cell preparations are stable, making them strong candidates for use in quality assessment programs in resource-limited settings [7], [8], [9]. SWBPs have demonstrated their utility for internal quality control monitoring as well as serving as testing panel material for quality assessment activities [10], [11], [12].

Over the past decade several new SWBPs have reached the market. Although all SWBPs are similar in respect to their cell types and cell subset components, some products are recommended for a specific brand of instrument and it is unclear if there is a single stabilised product compatible with the current array of commonly used CD4 T-cell counting platforms.

The objective of this study was to evaluate the compatibility of seven commercial SWBPs with currently utilised CD4 T-cell counting platforms and identify products compatible across the largest number of platforms for use as a quality assessment testing panel. Seven SWBPs (*Immuno-Trol (Beckman Coulter, Miami, FL)*, *CD-Chex Plus*, *CD-Chex Plus BC*, *CD4 Count (Streck Laboratories, Omaha, NE)*, *Multi-Check (BD Biosciences, San Jose, CA)*, *StatusFlow (R&D Systems, Minneapolis, MN)*, *CytoFix (Cytomark, Buckingham, UK*) with differing levels of target CD4 T-cells were tested on seven enumeration platforms (*FacsCalibur*, *FacsCount*, *Epics-XL*, *Guava PCA*, *CyFlow Counter*, *Pima*, *PointCare Now*).

## Material

### Stabilized whole blood products and CD4 platforms

Seven SWBPs (Table 1) with high and low level of CD4 T-cells were tested using thirteen reagent combinations, including a standardised reference method [13], on seven enumeration platforms (Table 2). This assessment took place between 2009 and 2011. The scope of the study, which combined multiple CD4 platforms and SWBPs, could not be achieved with a single lot of product. SWBPs were used at early stages of shelf-life where possible throughout the study.

**Table 1:** List of stabilized whole blood products tested.

Product Name	Company	Mean CD4 Level*	
		% ± SD	Count ± SD (cells/µL)
Immuno-Trol Cells	Beckman Coulter	48.4±0.3	635±14
Immuno-Trol Low Cells		18.0±0.6	156±11
CD-Chex Plus	Streck	48.8±0.7	1192±17
CD-Chex Plus CD4 Low		11.1±0.3	185±5
CD-Chex Plus BC		47.4±0.6	1185±28
CD-Chex Plus BC low		11.1±0.4	157±7
CD4 Count Normal		45.5±0.8	1114±26
CD4 Count Low		11.5±0.4	156±6
Multi-Check CD4 Control	BD Biosciences	47.6±0.6	702±20
Multi-Check CD4 low Control		13.1±0.6	137±7
StatusFlow	R&D Systems	50.6±0.6	864±28
StatusFlow Low		13.5±0.3	142±2
CytoFix CD4 Normal	Cytomark	52.6±0.5	646±5
CytoFix CD4 low		12.1±0.7	235±11

*Obtained by the reference method.

**Table 2:** List of CD4 enumeration platforms and antibody reagents tested with commercial stabilized whole blood products.

CD4 platform	Technology	Reagent	MAb combination	CD4	
				%	cells/µl
**FacsCalibur**	**Reference Method**	**MultiTest Reagent**	**CD3FITC/CD8PE/CD45PerCP/CD4APC**	✓	✓
**BD FACSCalibur [BD BioSciences, US]**	**a**	**Multi Test Reagent MultiSet**	**CD3FITC/CD8PE/CD45PerCP/CD4APC**	✓	✓
	**b**	**Tritest Reagent MultiSet**	**CD3FITC/CD4PE/CD45PerCP**	✓	✓
	**c**	**Tritest Reagent MultiSet**	**CD4FITC/CD8PE/CD3PerCP**		✓
**BD FACSCount [BD BioSciences, US]**	**d**	**BD FACSCount Reagent Kit**	**CD3PE-Cy5/CD4PE CD3PE-Cy5/CD8PE**		✓
	**e**	**BD FACSCount CD4 Reagent Kit**	**Lym/CD4PE**	✓	✓
**COULTER EPICS XL-MCL [Beckman Coulter, US]**	**f**	**CYTO-STAT tetraCHROME 4 Color reagent**	**CD45FITC/CD4RD1/CD8ECD/CD3PC5**	✓	✓
**Guava PCA [Millipore, US]**	**g**	**Guava Express CD3/CD4 Reagent Kit**	**CD3PE-Cy5/CD4PE**		✓
	**h**	**Guava Auto CD4/CD4% Kit**	**Lym PE-Cy5/CD4PE**	✓	✓
**CyFlow Counter [Partec, Germany]**	**i**	**CD4 easy count**	**CD4 PE**		✓
	**j**	**CD4% easy count**	**CD4PE/CD45PE-Dy647**	✓	✓
**Alere PIMA analyser [Inverness Medical-Clondiag, Germany]**	**k**	**Pima CD4 Cartridge**	**CD3PE-Cy5/CD4PE**		✓
**PointCare ** ***NOW*** ** [PointCare, US]**	**l**	**CD4Now Gold**	**Anti CD4 coated colloidal gold particles**	✓	✓

## Method

### Assessment criteria

Compatibility of SWBPs with CD4 technologies and their suitability for EQA programs were evaluated based on two criteria: (1) accuracy and (2) stability.

### Accuracy

The first phase of the study consisted of evaluating if CD4 T-cell levels within stabilized whole blood products could be measured accurately on each enumeration platform. Each SWBP was prepared with all possible antibody reagents in triplicate according to the manufacturer's instructions and analyzed on each platform. Whenever software analysis offered automated and manual mode, the sample preparation was analysed first using automation and reanalyzed in a manual mode when optimization was necessary. All the respective manufacturer's recommended instrument setup and quality control procedures were followed. For each of the SWBPs, the mean and standard deviation (SD) were calculated from triplicate CD4 T-cell measurements and compared to the mean value obtained for that SWBP using a reference test method. As can be observed from Table 3 and Table 4, the variation of the means +/− SD for each product, on each platform was minimal.

**Table 3:** Mean ± Standard Deviation of CD4 T-cell absolute count measurements for each stabilized whole blood product on the different enumeration platforms.

SWPB	CD4 Level	FacsCalibur			FacsCount		Epics-XL	Guava		CyFlow Counter		Pima
		a	b	c	d	e	f	g	h	i	j	k
Multi-Check	High	*765±25	*753±3	*786±8	664±27	669±112	633±22	670±9	698±13	*768±7	*723±22	*632±38
	Low	*135±1	*134±8	*125±6	122±7	125±8	123±4	131±7	126±2	*137±4	*133±6	*118±8
StatusFlow	High	1045±35	1027±34	1013±18	961±15	950±23	913±11	981±49	987±14	927±10	856±19	*718±37
	Low	184±8	177±8	191±10	173±6	171±6	160±5	148±4	176±5	156±2	153±6	*130±7
CD-Chex Plus	High	1283±42	1292±8	1343±47	NM	1274±43	1235±4	1385±35	1312±5	*1198±9	*1146±40	*1202±113
	Low	188±12	186±5	229±6	NM	189±3	171±6	189±5	205±5	*189±8	*174±12	*190±17
CD-Chex Plus BC	High	1486±58	1500±63	1485±52	1109±64	1434±57	1366±9	1588±43	1508±47	*986±6	*960±9	*1025±88
	Low	175±13	170±12	245±1	153±8	169±1	184±11	214±35	194±13	*138±2	*120±7	*159±13
CD4 Count	High	1098±26	1110±19	1156±54	1095±44	1067±24	1100±64	1226±72	1152±66	*1044±6	*978±25	*1093±49
	Low	176±4	178±8	180±2	180±23	183±2	189±4	191±9	193±10	*141±3	*132±6	*129±13
Immuno-Trol	High	563±1	559±18	596±16	596±14	626±34	570±14	643±5	616±18	*678±5	*630±12	*653±10
	Low	167±10	184±5	180±1	208±30	193±33	184±4	188±8	189±5	*167±9	*132±2	*159±10
CytoFix	High	685±23	672±11	656±21	649±11	673±2	549±19	808±10	679±18	583±13	598±23	614±36
	Low	253±17	239±15	273±8	241±14	NM	239±5	243±6	255±1	225±15	204±11	271±31

* = multiple lots combined.

NM = not measurable.

**Table 4:** Mean ± Standard Deviation of CD4 T-cell percentage measurements for each stabilized whole blood product on the different enumeration platforms.

SWPB	CD4 Level	FacsCalibur		FacsCount	Epics-XL	Guava	CyFlow Counter
		a	b	e	f	h	j
Multi-Check	High	43.9±0.5	43.4±0.4	42.5±0.7	41.6±0.3	45.9±0.7	*48.5±0.1
	Low	12.2±0.4	12.0±0.1	11.4±0.3	12.8±0.1	14.3±0.5	*14.0±0.5
StatusFlow	High	51.3±0.4	51.6±0.3	48.9±0.6	50.8±0.5	55.1±1.2	52.0±0.6
	Low	15.6±0.5	15.3±0.3	13.9±0.5	15.1±0.2	17.5±0.2	15.1±0.5
CD-Chex Plus	High	47.6±0.6	48.1±0.8	NM	46.4±0.5	51.8±1.6	*46.3±0.5
	Low	10.0±0.3	10.3±0.4	NM	9.8±0.3	12.8±0.1	*10.0±0.3
CD-Chex Plus BC	High	44.9±0.8	44.7±0.5	42.3±0.5	43.9±1.3	47.3±1.5	*47.3±0.7
	Low	7.2±0.3	6.9±0.4	5.8±0.1	7.4±0.7	8.6±0.5	*10.4±0.2
CD4 Count	High	40.8±0.7	41.9±0.4	39.9±0.4	40.0±0.9	44.0±0.3	*41.3±0.6
	Low	9.3±0.3	9.8±0.5	9.2±0.2	10.0±0.1	11.1±0.3	*10.1±0.2
Immuno-Trol	High	48.0±1.4	46.7±1.1	38.7±0.3	49.2±0.6	55.4±0.8	*42.0±0.7
	Low	16.8±1.1	17.7±0.9	15.3±0.8	18.1±0.3	21.1±0.5	*12.8±0.3
CytoFix	High	52.6±0.6	52.7±0.6	49.2±0.8	51.2±1.1	57.0±1.8	50.3±0.4
	Low	13.2±0.9	12.0±0.0	8.6±0.3	11.7±0.1	16.0±0.2	11.0±0.2

* = multiple lots combined.

NM = not measurable.

The reference test method is a universal template for single platform T-cell enumeration previously evaluated for a wide array of instruments and immunophenotyping settings within the Canadian Clinical Trial Network laboratories [13]. This method uses a double anchor gating strategy based on two cell lineage specific markers (CD45 and CD3). Samples were prepared as follows: 100 µl of SWBPs were incubated with 20 µl of BD MultiTest cocktail reagent (BD Biosciences) CD3FITC/CD8PE/CD45PerCP/CD4APC for 10 minutes at room temperature. SWBPs were then lysed using Immuno-Prep reagent (Beckman Coulter). Finally, 500 µl of 2% PFA was added followed by 100 µl of Flow-Count fluorospheres (Beckman Coulter). Preparations were acquired within 2 hours on a FacsCalibur (BD Biosciences, San Jose, CA) using BD CellQuest Pro software. The reference test method has been used since 2002, by the National Laboratory for HIV Immunology of the Public Health Agency of Canada which is certified by United States National Institute of Allergy and Infectious Diseases (NIAID) CD4 Immunology Quality Assessment Program (IQAP, https://iqa.center.duke.edu). Additionally, the reference method has been used by the National Laboratory for HIV Immunology during their participation in the external quality assurance programs; UK-NEQAS for Leucocyte Immunophenotyping Program (www.ukneqasli.co.uk) and Flow Cytometry: CD34+ Stem Cell Enumeration Program (www.wiv-isp.be) [8], [13].

Accuracy was established by dividing the mean CD4 count of each SWBP measured on each enumeration platform by their respective mean CD4 count obtained with the reference method. A ratio of 1 indicated that the CD4 counts were identical to the reference values. A ratio of less than 0.85 or greater than 1.15 was identified as not acceptable as determined by the largest expected inter-assay performance of CD4 technologies established by the WHO Prequalification of Diagnostics Programmes_PQDx [14]. For CD4 percentages, the absolute difference (residual) between each technology and reference method was measured. A residual value of ±3.0 or less was set for acceptability [15]. To facilitate interpretation, a binary scoring system was introduced to assess the overall performance. Products with CD4 measurements falling within, or falling outside limits were assigned a score of 1 or 0 respectively. Products that could not be measured by the technology were identified as not-measurable (NM) and assigned a score of 0.

### Stability

The second phase of the study consisted of evaluating the stability of SWBPs that satisfied accuracy criteria for relative and absolute counts on the largest number of enumeration platforms. Stability was determined based on the capacity of a preparation to sustain sub-optimal temperature environment to meet challenges related to transport and storage of specimens under extreme conditions observed in sub-Saharan Africa. SWBPs were first split into aliquots in order to dedicate a single aliquot for each time point. Aliquots were stored at room temperature (21–23°C) for testing at days 7, 10, 14 and 18 and at 37°C for testing at days 1, 2 and 3. Each product was prepared in triplicate and mean values were compared to the measurement of the product stored at the optimal temperature (4°C) and tested on day 0, time of initiation of the stability study. The acceptability criteria for stability were set using the same limits as determined for accuracy. Thus, the product was considered stable as long as the measurements fell within these limits.

Stability was evaluated using the following reagent kits: the MultiTest Reagent on the FacsCalibur, the FACSCount Reagent kit and FACSCount CD4 Reagent kit on the FACSCount (BD Biosciences, San Jose, CA), the Guava Express CD3/CD4 Reagent kit on the Guava PCA (EMD Millipore, Billerica, MA), the CD4 easy count and the CD4% easy count kit on the CyFlow Counter (Partec, Münster, Germany) and the Pima CD4 cartridge kit on the Alere PIMA analyser (Alere Technologies, Jena, Germany).

## Results

### Accuracy

To determine the cross platform accuracy of the seven SWBPs the accuracy of CD4 T cell absolute counts and percentages were measured using the described reagents on their respective enumeration platforms. SWBPs tested on the PointCare Now platform in the “patient” mode were not measurable for percentage or absolute CD4 counts [16], thus testing on this platform was terminated at this stage of the study.

**Figure 1:**
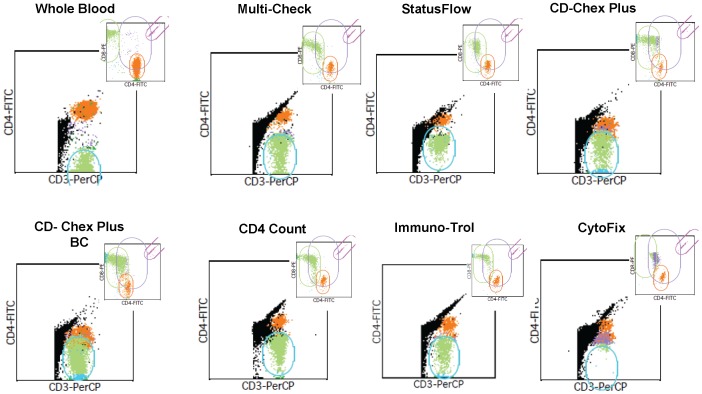
FacsCalibur Multiset analysis of stabilized whole blood products (SWBPs). Low CD4 level SWBP and fresh whole blood stained with CD4FITC/CD8PE/CD3PerCP antibody combination are shown. Two dot plots are shown for each analysis: CD3×CD4 with attractor gate on CD3+4− cells cluster; CD4×CD8 (upper right corner) with attractor gate on beads, CD4, CD8 and double positive CD4+8+ cells cluster.

For absolute count measurements, we found with the following exceptions that the majority of the seven SWBPs passed the accuracy test (Table 5). *CD-Chex Plus (High and Low)* was not measurable on the FacsCount when the FacsCount reagent kit was used; *CytoFix*-*Low* was not measurable on the FacsCount when the FacsCount CD4 reagent kit was used. Accuracy failed with *CD-Chex Plus-Low*, *CD-Chex plus BC-Low* and *CytoFix-Low* with differences greater than 15% as compared to the reference values on the FacsCalibur using MultiSet software with the CD4/CD8/CD3 combination. Figure 1 illustrates the MultiSet analysis of SWBPs (low CD4 level) on the FacsCalibur using the CD4/CD8/CD3 TriTest reagent. Compared to fresh whole blood, resolution between CD3+4− and CD3+4+ cells populations was lower for all products. Poor resolution was also observed with *CD-Chex Plus*, *CD-Chex Plus BC* and *CytoFix*. *CD-Chex Plus BC (High and Low)* and *CytoFix-High* failed on the Guava PCA platform using the Guava Express CD3/CD4 reagent kit. *CD-Chex Plus-Low* failed on Guava PCA using the CD4/CD4% reagent. *CD-Chex Plus BC-Low* and *Immuno-Trol-Low* failed on CyFlow Counter when the CD4% easy count kit was used. Thus, *Multi-Check*, *StatusFlow*, and *CD4 Count* show best scoring performance for both high and low CD4 level preparations.

**Table 5:** Ratios of mean value obtained for each stabilized whole blood product (SWBP) over mean reference value from CD4 absolute count measurements.

SWPB	CD4 Level	FacsCalibur			FacsCount		Epics-XL	Guava		CyFlow Counter		Pima	Score
		a	b	c	d	e	f	g	h	i	j	k	n/11
Multi-Check	High	1.09	1.07	1.12	1.07	1.08	1.02	1.08	1.12	0.95	0.90	0.90	**11**
	Low	1.10	1.10	1.02	1.01	1.03	1.02	1.08	1.04	0.90	0.87	0.96	**11**
StatusFlow	High	1.10	1.08	1.07	1.01	1.00	0.96	1.03	1.04	0.98	0.90	0.92	**11**
	Low	1.08	1.03	1.12	1.01	1.00	0.94	0.87	1.03	0.91	0.90	1.15	**11**
CD-Chex Plus	High	1.05	1.06	1.10	NM	1.07	1.01	1.14	1.08	1.01	0.97	1.03	**10**
	Low	1.09	1.08	*1.33*	NM	1.08	0.99	1.10	*1.17*	1.08	0.99	0.91	**8**
CD-Chex Plus BC	High	1.09	1.10	1.09	1.02	1.05	1.00	*1.16*	1.10	0.91	0.89	0.93	**10**
	Low	1.02	0.99	*1.43*	1.02	0.99	1.08	*1.25*	1.13	0.92	*0.80*	1.04	**8**
CD4 Count	High	1.00	1.01	1.05	1.00	0.97	1.00	1.12	1.05	0.97	0.91	0.92	**11**
	Low	1.02	1.03	1.04	1.04	1.05	1.09	1.10	1.12	0.93	0.87	1.01	**11**
Immuno-Trol	High	0.94	0.93	0.99	0.99	1.04	0.95	1.07	1.03	1.01	0.94	0.98	**11**
	Low	0.88	0.97	0.95	1.10	1.02	0.97	0.99	0.99	1.07	*0.84*	1.01	**10**
CytoFix	High	1.06	1.04	1.02	1.00	1.04	0.85	*1.25*	1.05	0.90	0.93	0.95	**10**
	Low	1.08	1.02	*1.16*	1.03	NM	1.02	1.03	1.09	0.96	0.87	1.15	**9**

Technologies “a–k” are detailed in table 2.

Italic values = values out of range.

NM = not measurable.

Score = total number of values within range.

**Figure 2:**
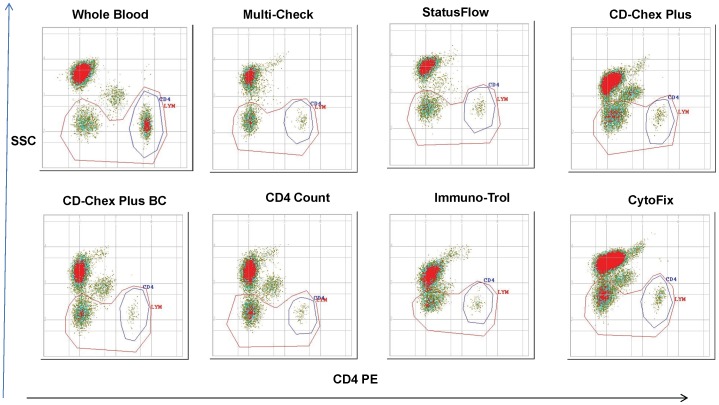
Analysis of Low CD4 level SWBPs on CyFlow. Stabilized whole blood products (low CD4 level) and fresh whole blood stained with CD4% easy count on CyFlow Counter are shown. Each CD4×SSC dot plot displays two gates: (1) “CD4” gate set around CD4 lymphocytes cluster and (2) “LYM” gate set around all lymphocytes.

We next assessed accuracy using CD4 T cell percentages, and found that again, the majority of SWBPs passed accuracy using CD4 T cell percentages (Table 6). However, accuracy failed with *Immuno-Trol (High and Low)*, *CD-Chex Plus BC-High*, and *CytoFix (High and Low)* with residual values >3.0 using the FACSCount CD4 reagent kit and *CD-Chex Plus (High and Low)* was not measurable. *Immuno-Trol-High* and *CytoF*ix *(High and Low)* failed on the Guava PCA using the Guava Auto CD4/CD4% kit. *Immuno-Trol (High and Low)* failed on the CyFlow Counter using the CD4% easy count kit. Figure 2 illustrates the analysis of SWBPs (low level) on the CyFlow Counter using the CD4% easy count reagent. The CD4×SSC dot plots displayed the CD4 and the lymphocyte gate. The resolution between CD4− lymphocyte and monocytes is critical for reliable gating for measurements of lymphocyte percentages. The resolution observed with *Immuno-Trol* was poor which increased the level of difficulty to draw a reliable gate and obtain high lymphocyte recovery and low monocyte contaminants. Thus, *Multi-Check*, *StatusFlow* and *CD4 Count* showed best accuracy and performance for both high and low CD4 percentage preparations on six of the platforms tested.

**Table 6:** Residual values obtained for each stabilized whole blood product (SWBP) from CD4 percentages measurements.

SWPB	% CD4	FacsCalibur		FacsCount	Epics-XL	Guava	CyFlow Counter	Score
Residual	level	a	b	e	f	h	j	n/6
Multi-Check	High	−0.5	−1.0	−1.9	−2.8	1.5	−0.1	**6**
	Low	−1.2	−1.3	−1.9	−0.5	0.9	0.8	**6**
StatusFlow	High	−0.4	0.1	−2.7	−0.9	3.5	0.4	**6**
	Low	0.3	0.0	−1.4	−0.2	2.2	−0.2	**6**
CD-Chex Plus	High	−1.6	−1.2	NM	−2.8	2.5	−1.9	**5**
	Low	−0.4	−0.2	NM	−0.7	2.3	−0.5	**5**
CD-Chex Plus BC	High	−0.9	−1.1	*−3.4*	−1.8	1.6	−1.3	**5**
	Low	−0.2	−0.5	−1.7	0.0	1.2	−0.4	**6**
CD4 Count	High	−1.1	0.1	−1.9	−1.8	2.2	−1.0	**6**
	Low	−0.5	0.0	−0.6	0.2	1.3	−0.1	**6**
Immuno-Trol	High	−1.6	−2.9	*−10.9*	−0.4	*5.8*	*−5.1*	**3**
	Low	−1.6	−0.8	*−3.1*	−0.3	2.7	*−4.0*	**4**
CytoFix	High	0.0	0.1	*−3.4*	−1.4	*4.4*	−2.2	**4**
	Low	1.0	−0.1	*−3.5*	−0.4	*3.9*	−1.2	**4**

Technologies “a–j” are detailed in table 2.

Italic values = values out of range.

NM = not measurable.

Score = number of values within the range.

In summary, three SWBPs (*Multi-Check*, *StatusFlow* and *CD4 Count*) were found to have the highest degree of accuracy for both absolute CD4 T cell count and percentages on the largest number of platforms examined, and were further assessed for stability.

### Stability

Stability of the three most compatible products was assessed at room temperature and at 37°C by examining accuracy of absolute T cell counts and percentage at both high and low CD4 levels (Table 7).

**Table 7:** Stability of *Multi-Check*, *StatusFlow* and *CD4 Count* stabilized whole blood products (SWBPs) under different conditions on various platforms.

			Lymphocyte percentages				Absolute counts			
			Room Temperature		37°C		Room Temperature		37°C	
	Technology	SWBP	High	Low	High	Low	High	Low	High	Low
			Days				Days			
FacsCalibur	a	*Multi-Check*	18	18	1	2	18	18	2	1
		*StatusFlow*	18	18	1	2	18	18	2	2
		*CD4 Count*	18	18	3	3	18	18	3	3
FacsCount	d	*Multi-Check*					18	18	1	1
		*StatusFlow*					18	18	1	1
		*CD4 Count*					18	18	3	3
	e	*Multi-Check*	14	18	1	2	14	18	1	2
		*StatusFlow*	18	18	1	2	18	18	1	2
		*CD4 Count*	18	18	3	3	18	18	3	3
CyFlow Counter	i	*Multi-Check*					18	18	3	3
		*StatusFlow*					18	18	3	2
		*CD4 Count*					18	18	3	3
	j	*Multi-Check*	18	18	2	3	18	18	3	3
		*StatusFlow*	18	18	3	3	18	18	3	1
		*CD4 Count*	18	18	3	3	18	18	3	3
Guava PCA	g	*Multi-Check*					18	18	<1	<1
		*StatusFlow*					18	18	<1	<1
		*CD4 Count*					18	18	3	3
Alere Pima analyser	k	*Multi-Check*					18	14	2	3
		*StatusFlow*					18	10	3	2
		*CD4 Count*					18	18	3	3

Based on CD4 T cell absolute count measurements, all products were stable for 18 days when stored at room temperature, with the exception of *Multi-Check-High* on FacsCount with the CD4 reagent kit which was stable up to 14 days, *Multi-Check-Low* and *StatusFlow-Low* on Pima which were stable up to 14 and 10 days respectively. When measuring CD4 T cell percentage, the SWBPs stored at room temperature were stable up to 18 days on all platforms with the exception of *Multi-Check*-*High* measured on the FacsCount, using the FACSCount CD4 Reagent kit which again was stable up to 14 days.

For the absolute count measurements of products stored at 37°C, *CD4 Count (High and Low)* was stable for 3 days on all enumeration platforms tested. *Multi-Check (High and Low)* were stable for 3 days when tested on Cyflow Counter with both reagent kits. The stability of *Multi-Check (High and Low)* was up to 2 and 3 days respectively when tested on Pima. *StatusFlow (High and Low)* were stable for 3 and 2 days respectively when tested on Pima and on CyFlow Counter with CD4 easy reagent. *Multi-Check (High and Low)* and *StatusFlow (High and Low)* could not be measured accurately on Guava PCA when stored at 37°C. *Multi-Check (High and Low)* and *StatusFlow (High and Low)* were stable for 1 day when assessed on the FacsCount using the FACSCount reagent kit. *Multi-Check (High and Low)* was stable for 2 and 1 day respectively when tested on the FacsCalibur while *StatusFlow* (*High and Low*) showed a 2-day stability. *Multi-Check-Low* and *StatusFlow-Low* were stable for 2 days while *Multi-Check-High* and *StatusFlow-High* were only stable for 1 day when tested on the FACSCount using the FACSCount CD4 reagent kit. Finally, *StatusFlow (High and Low)* were stable for 3 and 1 day respectively when tested on the CyflowCounter using CD4% easy count.

Based on CD4 T cell percentages measurements at 37°C, *CD4 Count (High and Low)* was stable on the FacsCalibur, the FacsCount and the CyFlow Counter for 3 days. Stability of high and low SWBPs was different between the other two products. Low CD4 level preparations of *Multi-Check* and *StatusFlow* were more stable than the high level samples on both the FacsCalibur and FacsCount. *Multi-Check* and *StatusFlow* were stable for 3 days when tested on the Cyflow Counter with the exception of *Multi -Check-High* which was stable only up to 2 days.

**Figure 3:**
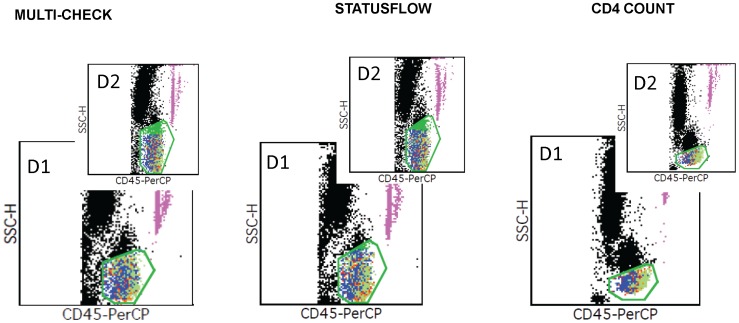
FacsCalibur Multiset analysis of different SWBPs. *Multi-Check*, *StatusFlow* and *CD4 Count* (low CD4 level) were prepared with MultiTest reagent CD3FITC/CD8PE/CD45PerCP/CD4APC on product incubated for 1 (D1) and 2 (D2) days at 37°C. Analysis displayed CD45×SSC dot plots with automated CD45 gates.

**Figure 4:**
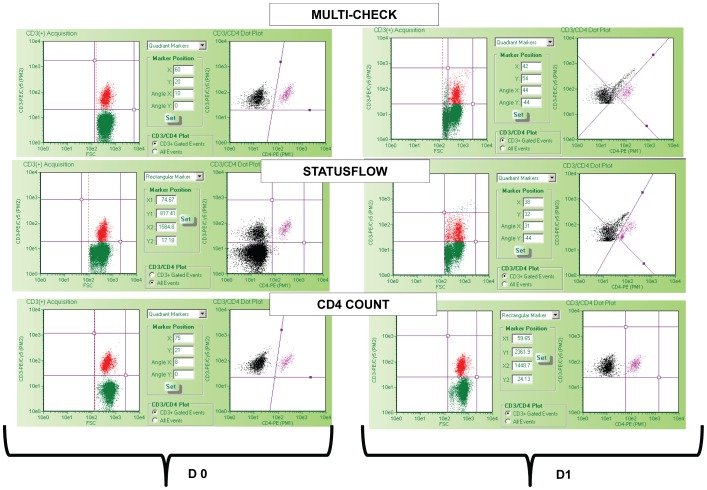
Guava PCA analysis of of different SWBPS. *Multi-Check*, *StatusFlow* and *CD4 Count* (low CD4 level) were prepared using the CD3/CD4 reagent kit on product stored at 4°C (D0) and products stored for 1 day at 37°C (D1). Analysis required first setting cursors around the CD3 cells population FSC×CD3 PECy5 dot plot and then isolating the CD4 positive cells cluster on CD4PE×CD3PECy5.

Incubation of SWBPs at suboptimal temperatures triggers sample degradation. Morphology and spectral properties may be lost rapidly. Testing of *Multi-Check* and *StatusFlow* products on the FacsCalibur was not continued beyond 2 days due to the inability to objectively gate the lymphocyte population as illustrated in Figure 3. The cursors placement around CD3 and CD4 cells clusters on Guava PCA was also challenging with *StatusFlow and Multi-Check* incubated a single day at 37°C, increasing the risk for unreliable measurements (Figure 4).

In summary, *CD4 Count* was found to be stable at 37°C for 3 days and at room temperature for 18 days for both CD4 T cell absolute counts and percentages on all of the enumeration platforms tested.

## Discussion

This study evaluated the compatibility of commercial SWBPs with CD4 T-cell enumeration technologies to identify an acceptable testing panel for EQA programs such as QASI. Such an update is required for a quality management program to keep abreast with increasing technological diversity in the field of CD4 T cell enumeration.

Compatibility of SWBPs was assessed based on the accuracy of the measurements, and stability of the product under suboptimal storage conditions. Stabilized whole blood products are primarily intended as quality controls for leukocyte immunophenotyping. Their degrees of similarity with fresh whole blood as well as their long term stability properties constitute significant benefits for the implementation of external quality assessment programs. There are products developed and optimized by manufacturers for specific enumeration platforms which are expected to perform optimally under specified conditions. However, we hypothesized that some products could be cross-compatible across multiple platforms without compromising performance.

This study demonstrated that all of the SWBPs tested could be measured accurately on more than one platform. Three SWBPs, *CD4 count*, *StatusFlow* and *Multi- Check*, were compatible with all the platforms tested with the exception of the PointCare Now. Considering the high degree of CD4 technological heterogeneity, these products would be the most suitable for quality assessment programs.

To ensure quality testing, it is critical to perform internal daily quality control and enroll into an external quality assessment program to identify poor performance and bring correctives. Therefore, the compatibility of CD4 enumeration technologies with EQA panels is essential to build confidence in the accuracy of CD4 results in patient specimens.

Compared to fresh whole blood, light scatter and fluorescence characteristics of SWBPs generally affect the resolution between cell populations. The importance of resolving between different populations is critical to the ability of fully automated software to identify the cluster of interest and reliably gate the target population. Poor resolution will negatively impact the accuracy of measurement and may lead to testing failure, specifically with automated analysis algorithm unable to identify clusters for gating purposes. Limitations of fully automated platforms such as the FACSCount and the PIMA resulted in aborted analysis of some SWBPs. This was observed for both *CD-Chex Plus* and *CytoFix* that could not be measured with one or both reagent kits on the FACSCount. Platforms with manual gating mode and adjustable cursors offer the user more flexibility. Nevertheless, if the product shows poor resolution, it may be challenging to gate reliably on the cluster of interest to maintain high recovery and purity. This was observed on the CyFlow Counter using the percent reagent kit and on the FacsCalibur with MultiSet and the TriTest antibody combination. In these situations, gating is subjective and unreliable. SWBPs with high resolution such as *CD4 count*, *Multi-Check and StatusFlow* will perform best with automated software algorithm.

In general, analysis of SWBPs is more challenging than fresh whole blood due to their differences in morpho-spectral characteristics. While such undesirable properties may be perceived negatively from a user's point of view who is not accustomed to moving cursors or gates, it can be used by the EQA provider as a valuable training tool to improve user's ability to recognize the limitations of automated software algorithms.

Clinical specimens stressed by external variables such as environmental effects or processing delays have morpho-spectral characteristics similar to those of SWBPs and require the user to apply manual override to achieve correct analysis.

Stability of commercial SWBPs is optimal when stored at 4°C for 30 to 90 days which should guarantee ample time for delivery and testing. However, international EQA programs such as QASI requires products which can resist temperature fluctuation during long term shipment, transit, and often suboptimal storage conditions associated with delays in custom clearance. Maintaining product stability until reaching the testing site is critical. Specimens must not be exposed to extreme conditions because high temperatures could destroy cells and affect test results. This study showed variation in the degree of stability among the three products (*CD4 Count*, *StatusFlow*, *Multi-Check*) when stored at sub optimal temperatures. Sample storage at 37°C will impair morpho-spectral characteristics faster as compared to ambient temperature and thus increase the level of difficulty for gating. The *CD4 Count* product at low and high CD4 levels was found to be more stable and resistant to sub optimal conditions than *StatusFlow* and *Multi-Check* for both CD4 T-cell absolute count and percentage measurements. SWBPs tested under sub-optimal storage conditions may react differently depending upon where they are within their declared shelf life. For that reason, products were used at early stages of shelf-life where possible throughout the study. It is also possible that lot-to-lot variation may impact on the outcome. Such additional parameters were not within the scope of this assessment.

Programs like QASI are designed to assist countries with potential to implement their own national quality assessment program. This study can be of assistance to other EQA providers in the selection of a quality-testing panel. Although this study is dedicated to SWBPs, similar testing algorithms are applicable to preparations using commercial fixatives, a chemical that is added to whole blood to extend its stability. Thus, the suitability of a product to conduct an EQA program is primarily based on the degree of compatibility of control panel with the technological heterogeneity of the platforms used to enumerate CD4 T-cells. Geographical location of the clinical sites and environmental conditions will dictate the required SWBP robustness.

This study demonstrated the importance of assessing the level of compatibility of stabilized whole blood controls with different CD4 enumeration platforms of interest. There is a wide array of products with different characteristics which need to be tested under various routine clinical conditions. These processed products used to monitor EQA laboratory performance may not behave like fresh whole blood specimens and contribute to matrix effect such as biases and lead to inaccurate conclusion. Therefore, it is critical to select appropriate quality control panel to avoid inaccurate conclusion about laboratory performance.

In summary, this study demonstrated that *CD4 Count*, *StatusFlow*, and *Multi-Check* are the most suitable stabilized whole blood products for EQA across multiple CD4 testing platforms based on their accurate measurements of both absolute counts and lymphocyte percentages. This study also showed that *CD4 Count* was the most robust when stored at suboptimal storage condition, an asset for international quality assessment programs.
